# Exposure to cooking oil fumes and chronic bronchitis in nonsmoking women aged 40 years and over: a health-care based study

**DOI:** 10.1186/s12889-018-5146-x

**Published:** 2018-02-13

**Authors:** Huang-Chi Chen, Chia-Fang Wu, Inn-Wen Chong, Ming-Tsang Wu

**Affiliations:** 1 Department of Internal Medicine, Kaohsiung Municipal Hsiao-Kang Hospital, Kaohsiung Medical University, Kaohsiung, Taiwan; 2 Department of Public Health, College of Health Sciences, Kaohsiung Medical University, Kaohsiung, Taiwan; 3 Research Center for Environmental Medicine, Kaohsiung Medical University, Kaohsiung, Taiwan; 4 Division of Pulmonary and Critical Care Medicine, Department of Internal Medicine, Kaohsiung Medical University Hospital, Kaohsiung, Taiwan; 5 Faculty of Medicine, College of Medicine, Kaohsiung Medical University Hospital, Kaohsiung, Taiwan; 6 Department of Family Medicine, Kaohsiung Medical University Hospital, Kaohsiung Medical University, Kaohsiung, Taiwan

**Keywords:** Chronic bronchitis, Cooking oil fumes, Pulmonary function test, Nonsmoking women

## Abstract

**Background:**

Little is known about the effect of exposure to cooking oil fumes (COFs) on the development of non-malignant respiratory diseases in nonsmoking women. This study investigated the relationship between exposure to COFs and chronic bronchitis in female Taiwanese non-smokers.

**Methods:**

Searching the 1999 claims and registration records maintained by Taiwan’s National Health Insurance Program, we identified 1846 women aged 40 years or older diagnosed as having chronic bronchitis (ICD-9 code: 491) at least twice in 1999 as potential study cases and 4624 women who had no diagnosis of chronic bronchitis the same year as potential study controls. We visited randomly selected women from each group in their homes, interviewed to collect related data including cooking habits and kitchen characteristics, and them a spirometry to collect FEV1 and FVC data between 2000 and 2009.

**Results:**

After the exclusion of thirty smokers, the women were classified those with chronic bronchitis (*n* = 53), probable chronic bronchitis (*n* = 285), and no pulmonary disease (*n* = 306) based on physician diagnosis and American Thoracic Society criteria. Women who had cooked ≥ 21 times per week between the ages of 20 and 40 years old had a 4.73-fold higher risk of chronic bronchitis than those cooking < 14 times per week (95% CI = 1.65–13.53). Perceived kitchen smokiness was significantly associated with decreased FEV1 (− 137 ml, *p* = 0.021) and FEV1/FVC ratio (− 7.67%, *p* = 0.008).

**Conclusions:**

Exposure to COF may exacerbate the progression of chronic bronchitis in nonsmoking women.

**Electronic supplementary material:**

The online version of this article (10.1186/s12889-018-5146-x) contains supplementary material, which is available to authorized users.

## Background

Chronic obstructive pulmonary disease (COPD) was ranked by World Health Organization (WHO) as the third leading cause of death in 2012, causing 3.1 million deaths globally and 5.6% of all deaths worldwide [1]. In Taiwan, the prevalence rate for this disease exceeds 6 % (> 6.1%) in adults older than 40 years [2]. COPD is potentially irreversible and patients with this disease in Taiwan are estimated to spend more than 1.8 million US dollars per year of medical treatment for this disease [3].

COPD is traditionally more common in males because a much larger proportion of men smoked cigarettes, a known cause of COPD. However, due to increases in tobacco use by women in high-income countries and increased risk of indoor air pollution in low-income countries, that gap is beginning to close [4]. Still cigarette smoking is much less prevalent in women in Taiwan than in Caucasian women in other countries (3–4% vs. ~ 28%) [5, 6]. Thus, there may other factors besides smoking related to the development of COPD in Taiwanese women. One of our previous healthcare based studies, for example, found women exposed to second-hand smoke (SHS) were 3.65-fold more likely to have chronic bronchitis than non-exposed women in Taiwan [7], suggesting the possibility of other lifestyle or airborne factors.

Taiwanese women are customarily responsible for preparing the meals for their families. Taiwanese-style cooking often involves pre-heating oil to smoking point before adding the ingredients for stir-frying or deep-frying, most often over natural gas or electric burners. Previous studies have found several carcinogens, including polycyclic aromatic hydrocarbons (PAHs), aromatic amines, and nitro-polycyclic aromatic hydrocarbons, in cooking oil fumes (COFs) and in the kitchens of Chinese homes where women prepare food daily [8–11]. In addition to carcinogens, Taiwanese-style cooking can generate other irritating chemicals, such as 1,3-butadiene, formaldehyde, and aldehyde [12–14] which can affect the respiratory tract and lead to inflammation of the airway. Although exposure to COFs has been associated with risk for several cancers, including lung and cervix [15–19], its relationship to chronic non-cancerous respiratory diseases, such as chronic bronchitis, remains unclear. Thus, we sought to extend our previous healthcare study beyond effect of second-hand smoke and chronic bronchitis, this time focusing on COF. To do this, we tapped Taiwan’s 1999 National Health Insurance (NHI) Database and NHI Registration Database to identify women forty years old who had been residents of Kaohsiung City, Taiwan, for five years or more and who had a previous diagnosis of chronic bronchitis or probable chronic bronchitis and the same population of women who had not been diagnosed with this disease. The women were visited in their homes and administered a survey which included questions about cooking habits and kitchen characteristics and gave them a spirometry test to collect lung function data (FEV1 and FVC).

## Methods

### Study area and study population

This ongoing healthcare based study has been described in detail previously [7]. Briefly, the 1999 claims and registration records of women aged 40 years or older living in Kaohsiung City for five years or more were identified in the database of Taiwan’s Kao-Ping District National Health Insurance Bureau, a branch of Taiwan’s National Health Insurance Program [20]. Kaohsiung city is a heavily industrialized harbor city located on the southwestern coast of Taiwan (153.6 km^2^) (Additional file 1: Figure S1).

**Fig. 1:**
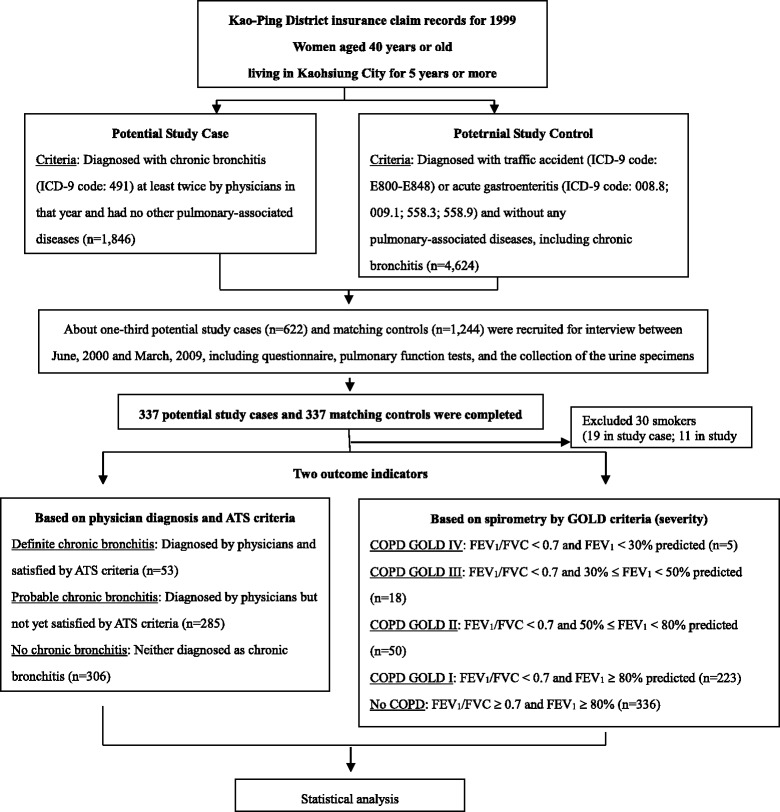
The study flowchart

A total of 221,965 women 40 years of age or older had lived in the city for five or more years and had made health insurance claims in 1999 [7]. Those diagnosed as having chronic bronchitis (ICD-9 code: 491) at least twice in 1999 were considered potential members of our study group (cases). Those who had made physician visits for traffic accidents (ICD-9 code: E800-E848) or acute gastroenteritis (ICD-9 code: 008.8; 009.1; 558.3; 558.9) the same year and who had never been diagnosed as having chronic bronchitis were considered potential study controls. Any case or control subject was excluded she had been diagnosed with other pulmonary-associated diseases, including asthma (ICD-9 code: 493), pulmonary tuberculosis (ICD-9 code: 011.9 or 010–018), bronchiectasis (ICD-9 code: 494), fibrotic cyst (ICD-9 code: 277), pulmonary tumor (ICD-9 code:162), emphysema (ICD-9 code: 492), extrinsic allergic alveolitis (ICD-9 code: 495), or chronic airway obstruction, not elsewhere classified (ICD-9 code: 496). After exclusion, we were left with 1846 potential study cases and 4624 potential controls (Fig. 1).

We randomly selected about one-third of 1846 potential study cases (*n* = 622) for subsequent in-person interviews and pulmonary function tests in their homes and randomly selected 1244 of the 4624 controls matching potential cases by age (within 3 years) and administrative area (Fig. 1). Once a study case was successfully recruited, interviewed and administered a pulmonary function test, one matched control was also immediately recruited, interviewed, and tested to adjust for the possible influence from external environmental hazards such as air pollution from large factories or traffic. In total, 337 cases and 337 controls completed the interview and pulmonary function tests between June 2000 and March 2009. This study was approved by IRB at Kaohsiung Medical University. All participants provided signed informed consent.

### Measurement of COF exposure

#### Questionnaire

Trained interviewers conducted personal face-to-face home interviews with the participants to collect epidemiologic data using a standardized questionnaire modified from the American Thoracic Society Division of Lung Disease Respiratory Symptom Questionnaire [2]. It included questions to collect demographic data including age, gender, educational level, ethnicity, and marital status as well as participant characteristics including family history of respiratory diseases, environmental chemical exposures in homes (second-hand smoke, COFs, mosquito coil burning and incense burning), and symptoms of chronic bronchitis.

We based our questions related to COF exposure on those we used in a previous community study of etiology of cervical intraepithelial neoplasm [19, 21]. Each participant was asked whether she cooked at home at least once a week between the ages of 20 and 40 years old. This question was also used in a study of Chinese food cooking and lung cancer risk in Taiwanese women [5]. The age range in this question is based on the assumption that Taiwanese women generally start cooking at the time of marriage (around 20 years old) and quit cooking at around the age of 40 years when their daughters-in-laws traditionally take over their duties. The daughter-in-law of the first son often moves into the house of her in-laws in an extended family in Taiwan. The age range in this question also takes into consideration the latent period between the time of exposure to COF and the occurrence of chronic diseases, such as COPD [19].

If a participant answered that she had cooked at home at least once a week, she was asked a detailed set of questions on cooking and ventilation conditions. These questions covered when she started cooking at home, how many years she had cooked, how many hours on average per day she stayed in the kitchen, what kind of fuels she used, whether there was a kitchen fume extractor installed in the home kitchen or not, how often the extractor was turned on while cooking, and whether she perceived the kitchen to be smoky or experienced any eye irritation while cooking. The interview lasted no more than 30 min on average. The interviewers could not be blinded to the health status of potential case/control. However, they only knew this activity was one part of health-promotion program and did not know the main hypothesis of this study.

#### Reliability of COF questions

One of our previous studies evaluated the reproducibility of the COF question items and found the test-retest reliabilities for most questions to have Kappa statistics (k) coefficients ranging between 0.69 and 0.75, indicating moderate to substantial agreement in one hundred study women [21]. In the current study, one to three months after the first interview, we re-asked 78 participants the question, “Between 20 and 40 years old, did you regularly cook at home (regularly, defined as at least once a week)?” The k coefficient was 0.51 (95% CI = 0.33–0.75), suggesting moderate agreement [22].

### Outcome measurement

#### Pulmonary function test

After each interview, each participant was administered a pulmonary function test using a portable spirometer (Micro Direct Inc. MSO3, Rochester, England). The measurement included forced expiratory volume in 1st second (FEV_1_) and forced vital capacity (FVC) [2]. Each participant performed the pulmonary function test three times and the highest values were recorded. None of the participants had smoked, eaten, or used any bronchodilators one hour before performing the pulmonary function test. All pulmonary function tests were administered following American Thoracic Society (ATS) guidelines [23]. To calculate the percent predicted values for lung function, we used predictive equations derived from Baldwin’s formula for FVC and Berglund’s formula for FEV1 [24, 25].

#### Chroni’c bronchitis related health status

We used two disease classification systems to categorize chronic bronchitis-related health status in our data analysis, as described in detail previously [7]. First, we classified all study subjects into three disease groups based on a physician’s diagnosis of chronic bronchitis at least twice in year 1999 and on ATS criteria for chronic bronchitis (the presence of cough and/or sputum production during the majority of days for at least three consecutive months in the previous two or more successive years) [23]. Subjects fulfilling both requirements were categorized as having “definite chronic bronchitis”, fulfilling one as having “probable chronic bronchitis”, and not fulfilling either as having “no pulmonary disease”. Second, using pulmonary function test data (FEV1 and FVC), we categorized COPD into various levels of severity as described previously using National Heart, Lung, and Blood Institute/World Health Organization GOLD criteria (Stages 0-IV) [26]: Stage 0, normal lung function (FEV_1_/FVC≥70% and FEV_1_≥80% predicted); Stage I, airflow mildly limited (FEV_1_/FVC < 70% and FEV_1_≥80% predicted); Stage II, airflow increasingly limited (FEV_1_/FVC < 70% and 50%≤ FEV_1_ < 80% predicted); Stage III (FEV_1_/FVC < 70% and 30%≤ FEV_1_ < 50% predicted) and finally Stage IV (FEV_1_/FVC < 70% and FEV_1_ < 30% predicted), severe airflow limitation. For this study, Stage 0 was grouped as “No COPD” and Stage I as “Mild COPD”. Because the sample size was small, Stages II-IV were categorized as “Moderate COPD” (Fig. 1).

### Statistical analysis

According to our previous published results [7], case and control response rates were 67.3% and 68.4%, respectively. The average age (±SD) (years) of case responders and non-responders were not significantly different (64.6 ± 9.6 vs. 63.2 ± 10.8, *p* = 0.11), and neither were age of the control responders and non-responders (64.6 ± 9.7 vs. 64.7 ± 11.2; *p* = 0.91). Because smoking is the main factor leading to chronic bronchitis [7], we excluded 19 smokers in the cases and 11 in the controls. Group characteristics were analyzed by the two disease classification systems and tested χ^2^ and Fisher’s exact tests, or ANOVA statistics when appropriate.

Polytomous logistic regressions were used evaluate the association between different COF variables and health status outcomes, adjusting for age, height, education (illiteracy vs. ≥ junior high school; primary school vs. ≥ junior high school), smoking status (SHS vs. non-smoker), incense burning (yes vs. no), and tea consumption (yes vs. no), all the potential covariates related to the risk of COPD, as suggested by our previous study [7]. Multiple linear regressions were used to investigate the relationship between the same individual variables and pulmonary function, either FEV_1_ or FEV1/FVC, both normally distributed. All statistical operations were performed using the SAS statistical package. Odds ratio (OR) and 95% confidence interval (CI) were calculated, and all *p-*values were two-sided.

## Results

After excluding thirty smokers (19 potential study cases and 11 controls), we reclassified the participants into three groups: “definite chronic bronchitis” (*n* = 53), “probable chronic bronchitis” (*n* = 285), and “no pulmonary disease” (*n* = 306) (Table 1; Additional file 1: Table S1). While none of the 326 controls had definite chronic bronchitis, twenty (6.1%) were classified as having probable chronic bronchitis (Additional file 1: Table S2). FEV1, smoking status, and tea consumption were significantly different across the groups before and after reclassification.

**Table 1:** Participant characteristics and pulmonary function test results of 644 non-smoking women categorized by physician diagnosed and ATS criteria

Variables	Definite chronic bronchitis (*N* = 53)	Probably chronic bronchitis (*N* = 285)	No pulmonary disease (*N* = 306)	*p*-value
Mean ± SD or N (%)				
Age (yrs)	61.9 ± 9.4	63.3 ± 8.8	63.3 ± 9.2	0.558
Height (cm)	155.1 ± 6.1	155.6 ± 5.7	155.1 ± 5.4	0.494
Weight (kg)	57.3 ± 13.0	58.6 ± 8.6	59.1 ± 8.6	0.392
BMI	23.8 ± 5.4	24.2 ± 3.3	24.6 ± 3.4	0.216
Pulmonary function test				
FEV_1_ (L)^a^	1.47 ± 0.45	1.53 ± 0.44	1.62 ± 0.47	0.012
FVC (L)^a^	1.85 ± 0.55	1.91 ± 0.55	1.92 ± 0.58	0.720
FEV_1_/FVC (%)^a^	80.5 ± 13.5	81.0 ± 14.7	86.0 ± 14.4	< 0.0001
Education levels^b^				
≥ junior high school	10 (18.9)	52 (18.3)	44 (14.4)	0.343
primary school	25 (47.2)	138 (48.4)	172 (56.4)	
illiteracy	18 (34.0)	95 (33.3)	89 (29.2)	
Smoking status				
Non-smoker	4 (7.6)	79 (27.7)	106 (34.6)	0.0002
Second-hand smoker	49 (92.5)	206 (72.3)	200 (65.4)	
Tea consumption				
No	50 (94.3)	241 (84.6)	236 (77.1)	0.003
Yes	3 (5.7)	44 (15.4)	70 (22.9)	
Burning incense				
No	15 (28.3)	112 (39.3)	120 (39.2)	0.291
Yes	38 (71.7)	173 (60.7)	186 (60.8)	

*ATS* American Thoracic Society; *BMI* body mass index; *FEV1* forced expiratory volume in 1st second; *FVC* forced vital capacity; *SD* standard deviation

^a^Missing data = 7 (3, 3, 1)

^b^Missing data = 1 (0, 0, 1)

As can be seen in Table 2, which analyzes the relationships between different COF exposure variables and risk of chronic bronchitis among non-smoking women, most of the participants cooked at least once a week between the ages 20 to 40 years (87.8% of those with no pulmonary disease and > 92% of those with probable and definite chronic bronchitis). The median age at which the women started cooking in a home kitchen was 22 years old. The greater the number of meals cooked per week, the higher the risk probable and definite chronic bronchitis, after adjusting for other covariates, including age, SHS status, height, education level, burning incense, and tea consumption. The risk for definite chronic bronchitis was highest in women who cooked ≥ 21 meals per week (adjusted OR = 4.73; 95% CI = 1.65–13.53) (Table 2; Additional file 1: Table S3).

**Table 2:** Relationships between variables of cooking oil fume exposure and the risk of chronic bronchitis among non-smoking women (*N* = 644)

Variables	No pulmonary disease (*N* = 306)	Probably chronic bronchitis (*N* = 285)			Definite chronic bronchitis (*N* = 53)		
	N (%)	N (%)	COR (95%CI)	AOR (95%CI)^*^	N (%)	COR (95%CI)	AOR (95%CI)^*^
Smoking status							
Non-smoker	106 (34.6)	79 (27.7)	1	1	4 (7.6)	1	1
Second-hand smoker	200 (65.4)	206 (72.3)	1.38 (0.97–1.96)	1.42 (0.99–2.03)	49 (92.5)	6.49 (2.28–18.48)	6.66 (2.31–19.20)
Cooked in the kitchen							
No	22 (7.2)	13 (4.6)	1	1	5 (9.4)	1	1
Yes	284 (92.8)	272 (95.4)	1.62 (0.80–3.82)	1.65 (0.80–3.43)	48 (90.6)	0.74 (0.27–2.06)	0.73 (0.22–2.43)
Excluding no cook							
Age stared cooking							
> 22 years	126 (44.4)	132 (48.5)	1	1	21 (43.8)	1	1
≤ 22 years	158 (55.6)	140 (51.5)	0.85 (0.61–1.18)	0.86 (0.61–1.23)	27 (56.3)	1.03 (0.55–1.90)	1.13 (0.58–2.20)
Meals per week							
1–13	85 (29.9)	55 (20.2)	1	1	5 (10.4)	1	1
14–20	94 (33.1)	84 (30.9)	1.38 (0.88–2.16)	1.52 (0.95–2.42)	14 (29.2)	2.53 (0.88–7.32)	2.71 (0.84–8.14)^§^
21–31	105 (37.0)	133 (48.9)	1.96 (1.28–2.99)	2.14 (1.36–3.36)^†^	29 (60.4)	4.69 (1.74–12.65)	4.73 (1.65–13.53)^†^
Fuel used for cooking^a^							
Gas/electric stove	179 (63.0)	152 (56.1)	1	1	28 (58.3)	1	1
Biomass fuels	67 (23.6)	68 (25.1)	1.20 (0.80–1.78)	1.39 (0.85–2.26)	12 (25.0)	1.15 (0.55–2.38)	1.15 (0.47–2.85)
Coal	38 (13.4)	51 (18.8)	1.58 (0.99–2.54)	1.83 (1.07–3.13)^‡^	8 (16.7)	1.35 (0.57–3.18)	1.36 (0.51–3.65)^‡^
Windows in the home kitchen^b^							
No	15 (5.3)	17 (6.4)	1	1	6 (13.6)	1	1
≥ 1	266 (94.7)	247 (93.6)	0.82 (0.40–1.68)	0.80 (0.38–1.68)	38 (86.4)	0.36 (0.13–0.98)	0.40 (0.14–1.16)
Ventilation in the home kitchen^c^							
Poor	14 (5.0)	9 (3.3)	1	1	6 (12.5)	1	1
Good	269 (95.1)	261 (96.7)	1.51 (0.64–3.55)	1.46 (0.61–3.50)	42 (87.5)	0.36 (0.13–1.00)	0.35 (0.12–1.04)
Installed fume extractor^d^							
No	122 (43.6)	128 (48.3)	1	1	25 (52.1)	1	1
Yes	158 (56.4)	137 (51.7)	0.83 (0.59–1.16)	0.75 (0.50–1.12)	23 (47.9)	0.71 (0.39–1.31)	0.69 (0.34–1.43)
Eye irritation during cooking^e^							
No	241 (96.0)	238 (96.0)	1	1	42 (93.3)	1	1
Yes	10 (4.0)	10 (4.0)	1.01 (0.41–2.48)	1.05 (0.42–2.62)	3 (6.7)	1.72 (0.46–6.52)	1.97 (0.48–8.01)
Smokiness during cooking^f^							
No	232 (92.1)	226 (91.1)	1	1	40 (88.9)	1	1
Yes	20 (7.9)	22 (8.9)	1.13 (0.60–2.13)	1.17 (0.61–2.24)	5 (11.1)	1.45 (0.52–4.09)	1.36 (0.46–4.00)

*AOR* adjusted OR; *COR* crude OR; *OR* odds ratio

^*^Adjusting for age, second hand smoke status, height, education level, burning incense, and tea consumption

^†^Trend test from 1 to 13, 14–20, to 21–31 meals per week in probably chronic bronchitis: *p* = 0.009; and in chronic bronchitis: *p* = 0.0023

^‡^Trend test from gas/electric stove, biomass fuels, to coal in probably chronic bronchitis: *p* = 0.025; and in chronic bronchitis: *p* = 0.544

^§^Trend test from no pulmonary disease, probably chronic bronchitis, to chronic bronchitis in 14–20 meals per week: *p* = 0.020; and in 21–31 meals per week: *p* <  0.0001

^a^Missing data, *n* = 1 (0,1,0). ^b^Missing data, *n* = 15 (3,8,4). ^c^Missing data, *n* = 3 (1,2,0). ^d^Missing data, *n* = 11 (4,7,0). ^e^Missing data, *n* = 60 (33,24,3). ^f^Missing data, *n* = 59 (32,24,3)

Applying the GOLD criteria, we found kitchen smokiness and eye irritation while cooking to be significantly associated with risk of moderate COPD and mild + moderate COPD, respectively (Table 3; Additional file 1: Table S4). Those who had cooked ≥ 21 meals per week were at relatively higher risk of mild or moderate COPD than those who had cooked < 14 meals per week, though significance was not reached (Table 3; Additional file 1: Table S4). With regard to pulmonary function, number of meals cooked per week and kitchen smokiness were negatively and significantly associated with FEV1 and FEV1/FVC ratio after adjustment (Table 4). For example, kitchen smokiness was significantly associated with the decreased FEV1 (− 137 ml, *p* = 0.021) and FEV1/FVC ratio (− 7.67%, *p* = 0.008) in non-smoking Taiwanese women. Good kitchen ventilation was significantly associated with higher FEV1/FVC ratio, but not FEV1.

**Table 3:** Relationships between variables of cooking oil fume exposure and the severity of COPD according to GOLD criteria (*N* = 632)

Variables	No COPD (*N* = 336)	Mild COPD (*N* = 223)			Moderate COPD (*N* = 73)		
	N (%)	N (%)	COR (95%CI)	AOR (95%CI)^*^	N (%)	COR (95%CI)	AOR (95%CI)^*^
Smoking status							
Non-smoker	108 (32.1)	66 (29.6)	1	1	11 (15.1)	1	1
Second-hand smoker	228 (67.9)	157 (70.4)	1.13 (0.78–1.63)	1.03 (0.70–1.52)	62 (84.9)	2.67 (1.35–5.27)	2.98 (1.47–6.01)
Cooked in the kitchen							
No	24 (7.1)	12 (5.4)	1	1	2 (2.7)	1	1
Daily	312 (92.9)	211 (94.6)	1.35 (0.66–2.76)	1.07 (0.51–2.27)	71 (97.3)	2.73 (0.63–11.82)	2.04 (0.46–9.18)
Excluding no cook							
Age stared cooking							
> 22 years	139 (44.6)	104 (49.3)	1	1	33 (46.5)	1	1
≤ 22 years	173 (55.5)	107 (50.7)	0.83 (0.58–1.17)	0.69 (0.47–1.00)	38 (53.5)	0.93 (0.55–1.55)	0.87 (0.49–1.52)
Meals per week^a^							
1–13	85 (27.2)	45 (21.3)	1	1	13 (18.3)	1	1
14–20	100 (32.1)	76 (36.0)	1.44 (0.90–2.29)	1.39 (0.86–2.25)	15 (21.1)	0.98 (0.44–2.18)	0.85 (0.37–1.94)
21–31	127 (40.7)	90 (42.7)	1.34 (0.85–2.10)	1.23 (0.77–1.98)	43 (60.6)	2.21 (1.12–4.36)	1.63 (0.80–3.33)
Fuel used for cooking^b^							
Gas/electric stove	194 (62.4)	125 (59.2)	1	1	33 (46.5)	1	1
Biomass fuels	74 (23.8)	52 (24.6)	1.09 (0.72–1.66)	0.89 (0.54–1.49)	21 (29.6)	1.67 (0.91–3.07)	0.87 (0.41–1.85)
Coal	43 (13.8)	34 (16.1)	1.23 (0.74–2.03)	1.12 (0.64–1.98)	17 (23.9)	2.32 (1.19–4.55)	1.36 (0.63–2.97)
Windows in the home kitchen^c^							
No	22 (7.2)	12 (5.9)	1	1	4 (5.7)	1	1
≥ 1	285 (92.8)	192 (94.1)	1.24 (0.60–2.56)	1.31 (0.62–2.77)	66 (94.3)	1.27 (0.43–3.82)	1.67 (0.54–5.20)
Ventilation in the home kitchen^d^							
Poor	14 (4.5)	8 (3.8)	1	1	7 (9.9)	1	1
Good	297 (95.5)	201 (96.2)	1.18 (0.49–2.88)	1.18 (0.48–2.94)	64 (91.0)	0.43 (0.17–1.11)	0.49 (0.19–1.30)
Installed fume extractor^e^							
No	131 (42.7)	99 (48.3)	1	1	39 (54.9)	1	1
Yes	176 (57.3)	106 (51.7)	0.80 (0.56–1.14)	0.85 (0.55–1.29)	32 (45.1)	0.61 (0.36–1.03)	1.01 (0.55–1.86)
Eye irritation during cooking^f^							
No	277 (97.5)	174 (93.6)	1	1	60 (93.8)	1	1
Yes	7 (2.5)	12 (6.5)	2.73 (1.05–7.06)	3.02 (1.14–8.00)	4 (6.3)	2.64 (0.75–9.30)	1.96 (0.54–7.17)
Smokiness during cooking^g^							
No	267 (93.7)	171 (91.9)	1	1	52 (81.3)	1	1
Yes	18 (6.3)	15 (8.1)	1.30 (0.64–2.65)	1.22 (0.59–2.53)	12 (18.8)	3.42 (1.56–7.53)	2.89 (1.28–6.55)

*AOR* adjusted OR, *COPD* chronic obstructive pulmonary diseases, *COR* crude OR, *GOLD* Global Initiative for Chronic Obstructive Lung Disease, *OR* odds ratio

^*^Adjusting for age, second hand smoke status, height, education level, burning incense and tea consumption

^a^Missing data, *n* = 10 (0,10,0). ^b^Missing data, *n* = 11 (1,10,0). ^c^Missing data, *n* = 13 (5,7,1). ^d^Missing data, *n* = 3 (1,2,0). ^e^Missing data, *n* = 11 (5,6,0). ^f^Missing data, *n* = 60 (28,25,7). ^g^Missing data, *n* = 59 (27,25,7)

**Table 4:** Relationships between variables of cooking oil fume exposure and pulmonary function (absolute FEV_1_ level and FEV_1_/FVC (%)) in multiple linear regression models

Variables			Adjusted analysis^*^			Adjusted analysis^*^	
	N	FEV1 (L) (mean ± SD)	β (SE)	*p*-value	FEV1/FVC (%) (mean ± SD)	β (SE)	*p*-value
Smoking status							
Non-smoker	187	1.57 ± 0.44	1	–	87.24 ± 13.00	1	–
Second-hand smoker	450	1.57 ± 0.47	−0.054 (0.033)	0.108	81.73 ± 15.05	−5.71 (1.27)	< 0.0001
Cooked in the kitchen							
No	39	1.70 ± 0.46	1	–	88.15 ± 11.97	1	–
Yes	598	1.56 ± 0.46	−0.07 (0.06)	0.270	83.03 ± 14.80	−4.84 (2.45)	0.048
Excluding no cook							
Age stared cooking							
> 22 years	278	1.58 ± 0.46	1	–	82.03 ± 14.56	1	–
≤ 22 years	320	1.55 ± 0.45	0.029 (0.032)	0.374	83.90 ± 14.98	1.46 (1.23)	0.235
Meals per week							
1–13	145	1.69 ± 0.47	1	–	84.98 ± 14.08	1	–
14–20	192	1.58 ± 0.43	−0.027 (0.042)	0.525	84.81 ± 12.84	0.15 (1.61)	0.927
21–31	261	1.47 ± 0.46	−0.065 (0.041)	0.111^†^	80.64 ± 16.19	−3.35 (1.55)	0.031^†^
Fuel used for cooking							
Gas/electric stove	354	1.69 ± 0.44	1	–	83.46 ± 13.17	1	–
Biomass fuels	147	1.38 ± 0.41	−0.036 (0.044)	0.414	82.56 ± 16.19	1.18 (1.70)	0.487
Coal	96	1.34 ± 0.42	−0.103 (0.048)	0.034^‡^	82.06 ± 18.06	0.60 (1.86)	0.746
Windows in the home kitchen							
No	38	1.59 ± 0.57	1	–	81.68 ± 16.11	1	–
≥ 1	546	1.56 ± 0.45	− 0.122 (0.064)	0.056	83.27 ± 14.75	0.75 (2.46)	0.760
Ventilation in the home kitchen							
Poor	29	1.42 ± 0.51	1	–	76.28 ± 16.45	1	–
Good	566	1.57 ± 0.46	0.103 (0.073)	0.156	83.41 ± 14.67	5.89 (2.77)	0.034
Installed fume extractor							
No	271	1.41 ± 0.42	1	–	81.91 ± 15.90	1	–
Yes	316	1.69 ± 0.45	0.041 (0.037)	0.261	83.89 ± 13.87	0.43 (1.41)	0.759
Eye irritation during cooking							
No	515	1.59 ± 0.45	1	–	82.92 ± 14.43	1	–
Yes	23	1.32 ± 0.62	−0.158 (0.082)	0.055	76.15 ± 22.16	−5.34 (3.12)	0.088
Smokiness during cooking							
No	493	1.59 ± 0.44	1	–	83.33 ± 13.96	1	–
Yes	46	1.41 ± 0.57	−0.137 (0.059)	0.021	75.07 ± 21.08	−7.67 (2.24)	0.001

^*^Adjusting for age, height, second-hand smoking status, education level, burning incense and tea consumption

^†^Trend test from 1 to 13, 14–20, to 21–31 meals per week in FEV1: *p* = 0.100; in FEV1/FVE(%): *p* = 0.016

^‡^Trend test from gas/electric stove, biomass fuels, to coal in FEV1: *p* = 0.037

## Discussion

This study found that the frequency of meals cooked per week and kitchen smokiness while cooking between the ages of 20- and 40-years-old to be the main COF determinants for progression of later chronic bronchitis and impaired lung function in non-smoking women in Taiwan and that good kitchen ventilation may reduce impairment of lung function.

COFs contain more than two hundred kinds of harmful particulates and gases, many of which have been identified as human carcinogens and environmental irritants [8–11, 27]. These chemicals include benzene, formaldehyde, 1,3-butadiene, aromatic amines, polycyclic aromatic hydrocarbons (PAHs) such as benzo[a]pyrene (B[a]P), benz[a]anthracene, and dibenz[a,h]anthracene, and acrolein, all known to cause not only mutagenicity and genotoxicity but also inflammatory or irritant reactions in the airways. With regard to genotoxicity, for example, in-vitro and human studies have reported 8-hydroxy-2′-deoxyguanosine causes dose-dependent increases in the levels of B[a]P 7,8-diol 9,10-epoxide N2-deoxyguanosine-DNA adducts and oxidative DNA damage when exposure to COF or fume extracts [28–30].

In addition to having carcinogenic effects, exposure to chemicals from fume condensates may cause airway injuries and inflammatory responses through their effect on oxidant and antioxidant imbalances and innate immunity impairment [31–33]. Wu et al.*,* investigating the chemical trans-trans-2,4-decadienal (t-t-2,4-DDE) detected in peanut oil fumes, found glutathione (GSH) content as well as the activities of antioxidative enzymes such as GSH reductase, GSH peroxidase and GSH S-transferase were reduced by the methanolic extract of oil fumes [34]. That study found that t-t-2,4-DDE produced superoxide anion, hydrogen peroxide, and hydroxyl radicals in a phosphate buffer (pH 7.4) and found that it induced intracellular reactive oxidative stress (ROS) in A-549 cells. Another study reported similar findings in oil fumes created by heating three common commercial cooking oils (soybean oil, sunflower oil, and lard) [35]. In one recent published paper, Peng and her colleagues, studying COF levels resulting from different kinds of cooking methods and uses of cooking oils, found that use of palm oil or rapeseed oil could reduce COF exposure, especially for long-chain aldehydes such as hexanal and t,t-2,4-DDE [36]. Tung et al. also reported that COF exposure induced cytokine expression (TGF*β*1) and oxidative stress in CL3 lung epithelial cells [37]. These findings suggest COF-induced inflammatory response in the airways may lead to respiratory symptoms or lung function impairment and contribute to development of chronic bronchitis.

Many epidemiological studies have reported an association between COF exposure and lung cancer risk [5, 15–18]. One recent meta-analysis summarized 13 articles including three population-based case-control studies and ten hospital-based case-control studies in nonsmoking Chinese women, totaling 3596 women with lung cancer and 6082 healthy controls [27]. That meta-analysis found the pooled estimates of risk ratio in fixed effects model and random effects model to be 1.74 (95% CI =1.57–1.94) and 2.11 (95% CI =1.54–2.89), respectively. Very few studies, however, have investigated the effect of COF exposure on non-malignant respiratory diseases [34, 38, 39]. Most of the studies that did study this effect focused on indoor smoke exposure when biomass fuels were used (e.g., wood, excrement, straw, etc.) while cooking [38, 39]. Dennis et al.*,* conducting a case-control study in Colombia, found the use of wood for cooking to be significantly associated with the development of obstructive airways disease (OR = 3.43; *p* <  0.001) [38]. Another population survey has also found biomass fuel use (especially wood) to be an important deteriorating factor for pulmonary function values, including FEV_1_, FEV_1_%, peak expiratory flow rate (PEFR) and mid-flow rate (defined as the forced expiratory flow (FEF) from 25% to 75% of the vital capacity) in females [39]. In one study of professional chefs, Svendsen and colleagues reported that female kitchen workers had higher prevalence rates of dyspnea (relative risk (RR) = 4.1; 95% CI = 2.7–6.3), serious dyspnea (RR = 2.9; 95% CI = 1.5–5.7)), and respiratory symptoms related to work (RR = 4.3; 95% CI = 2.7–6.7) compared to their female controls, suggesting that there is an association between exposure to cooking fumes and the development of respiratory diseases other than cancer in kitchen workers.

Cigarette smoking is a well-known major risk factor for both malignant and nonmalignant respiratory diseases. Indeed, in a previous study, we found that women who smoked and women who had been exposed to a lifetime of SHS were 24.81 times (95% CI: 5.78–106.38) and 3.65 times (95% CI: 1.19–11.26) more likely to have chronic bronchitis than those who had not been exposed to SHS [7]. In the current study, we also found that SHS exposure to be major contributor the development of chronic nonmalignant respiratory diseases among nonsmoking women. The adverse effects of COF exposure in this study were probably not as strong as those of SHS exposure (Table 2; Table 3). The reasons for this difference may be that (1) the concentrations of respiratory irritants maybe lower in COF chemicals than SHS chemicals or (2) the information about COF exposure we collected maybe not as accurate as that of SHS exposure. We found reproducibility of some COF exposure variables queried on our survey to have moderate agreement; thus, any misclassification would probably be random and cause an underestimation of the significance.

This study has several limitations. As mentioned above, data on COF exposure, including cooking activities, different kinds of oils, and ventilation systems etc., were collected by questionnaire. This would give rise to the possibility of recall bias, though that bias would likely be random and result in a null effect. For our outcomes of interest, the only objective measurement in this study was the pulmonary function test. Thus, there is a likelihood for some information bias in our subjective measurements. Another limitation is the occurrence of competing risk with cancer, since the previous studies have associated COF exposure with various cancers, especially lung cancer [19]. The age range at which malignant respiratory diseases occur is younger than the ages that nonmalignant respiratory usually occur. This might lead to a systematic underestimation of the prevalence of COPD in this population-based study. Another limitation is that we had no data on the participant’s past disease status, because our insurance health dataset had only the diagnostic codes recorded for that year (1999). This might lead to a random misclassification of outcome, though this possibility is reduced by fact that we also used ATS criteria to confirm original diagnoses reported by their physicians. Because this is an observational study and chronic bronchitis/COPD is a multi-factorial disease, other unmeasured confounders could have potentially affected our findings.

## Conclusion

In conclusion, the frequency of meals cooked per week and kitchen smokiness during cooking between the ages of 20- and 40-years-old are important contributing factors for chronic bronchitis and impaired lung function in nonsmoking Taiwanese women. Good kitchen ventilation may reduce the lung function impairment. Future studies may want to explore optimal design for kitchen ventilators to decrease the aggravation of COPD caused by COF exposure.

## Additional file

Additional file 1: **Table S1.** Distributions of demographic characteristics categorized by study cases and controls among non-smoking women (*N* = 644). **Table S2.** The distribution across our study subjects with different health statuses. **Table S3.** Relationships between major variable of cooking oil fume exposure and other covariates in non-cook women and the risk of chronic bronchitis among non-smoking women (*N* = 644). **Table S4.** Relationships between cooking and ventilation conditions in home kitchens between 20 and 40 years old and the severity of COPD according to GOLD criteria (*N* = 632). **Figure S1.** Study area. (DOCX 64 kb)
