# Supplementary material for: Exposure to cooking oil fumes and chronic bronchitis in nonsmoking women aged 40 years and over: a health-care based study
Source: BMC Public Health. 2018 Feb 13;18:246. doi: 10.1186/s12889-018-5146-x (PMC5812191; doi:10.1186/s12889-018-5146-x)
Supplement: Supplementary file 1 — Table S1. Distributions of demographic characteristics categorized by study cases and controls among non-smoking women (N = 644). Table S2. The distribution across our study subjects with different health statuses. Table S3. Relationships between major variable of cooking oil fume exposure and other covariates in non-cook women and the risk of chronic bronchitis among non-smoking women (N = 644). Table S4. Relationships between cooking and ventilation conditions in home kitchens between 20 and 40 years old and the severity of COPD according to GOLD criteria (N = 632). Figure S1. Study area. (DOCX 64 kb) [file 12889_2018_5146_MOESM1_ESM.docx]

**Supplementary Materials**

**Exposure to cooking oil fumes and chronic bronchitis in nonsmoking women aged 40 years and over: A health-care based study**

Huang-Chi Chen^1*^, Chia-Fang Wu^2,3*^, Inn-Wen Chong^4,5^, Ming-Tsang Wu^2,3,6^

*These authors contributed equally to this work.

**Legends**

**eTable 1. Distributions of demographic characteristics categorized by study cases and controls among non-smoking women (N=644).**

**eTable 2. The distribution across our study subjects with different health status (N=674).**

**eTable 3. Relationships between major variable of cooking oil fume exposure and other covariates in non-cook women and the risk of chronic bronchitis among non-smoking women (N=644).**

**eTable 4**. **Relationships between cooking and ventilation conditions in home kitchens in 20-40 years old status and the severity of COPD according to GOLD criteria (N=632).**

**eFigure 1. Study area. (Software: DIVA-GIS,** [**http://www.diva-gis.org**](http://www.diva-gis.org)**; QGIS, http://www.qgis.org/en/site/)**

**
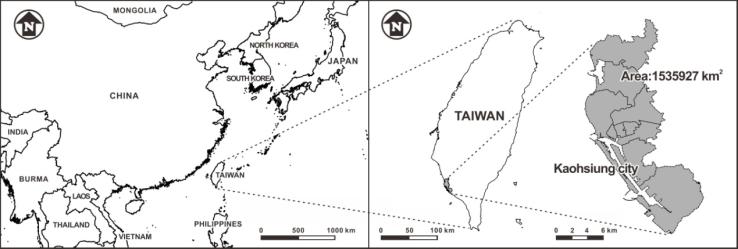
**

**eFigure 1. Study area. (Software: DIVA-GIS,** [**http://www.diva-gis.org**](http://www.diva-gis.org)**; QGIS, http://www.qgis.org/en/site/)**

The maps are created by a free GIS software, QGIS, and modified by CorelDraw X6.

| **eTable 1. Distributions of demographic characteristics categorized by study cases and controls among non-smoking women (N=644).** | | | |
| --- | --- | --- | --- |
| **Variables** | **Cases (N=318)** | **Controls (N=326)** | ***p*-value** |
|  | **Mean ± SD or N (%)** | |  |
| Age (years) | 63.1 ± 8.9 | 63.3 ± 9.2 | 0.858 |
| Height (cm)^*^ | 155.3 ± 5.7 | 155.3 ± 5.5 | 0.984 |
| Weight (kg)^†^ | 58.2 ± 9.5 | 59.2 ± 8.6 | 0.166 |
| BMI | 24.1 ± 3.7 | 24.5 ± 3.4 | 0.115 |
| FEV_1_ (l)^‡^ | 1.51 ± 0.44 | 1.63 ± 0.47 | 0.001 |
| FVC (l)^‡^ | 1.89 ± 0.55 | 1.92 ± 0.58 | 0.500 |
| Education levels |  |  |  |
| ≥ junior high school | 55 (17.3) | 51 (15.7) | 0.306 |
| primary school | 156 (49.1) | 179 (55.1) |  |
| illiteracy | 107 (33.7) | 95 (29.2) |  |
| Smoking status |  |  |  |
| Non-smoker | 78 (24.5) | 111 (34.1) | 0.008 |
| Second-hand smoker | 240 (75.5) | 215 (66.0) |  |
| Tea consumption |  |  |  |
| No | 277 (87.1) | 250 (76.7) | 0.0006 |
| Yes | 41 (12.9) | 76 (23.3) |  |
| Burning incense |  |  |  |
| No | 119 (37.4) | 128 (39.3) | 0.631 |
| Yes | 199 (62.6) | 198 (60.7) |  |
| Abbreviation: BMI = body mass index; FEV1 = forced expiratory volume in 1st second; FVC = forced vital capacity; SD = standard deviation.  ^*^Missing data=1 (1,0);  ^†^Missing data=5 (4,1) | | | |
| ^‡^Missing data=7 (6,1) | | | |
|  | | | |

**eTable 2. The distribution across our study subjects with different health status.**

| N(%) | Study controls  (n=337) | Study cases  (n=337) |
| --- | --- | --- |
| Based on physician diagnosis and ATS criteria | |  |
| No pulmonary disease  Probably chronic bronchitis  Chronic bronchitis | 316 (93.8%)  21 (6.2%)  0 | 0  278 (82.5%)  59 (17.5%) |
| Based on spirometry by GOLD criteria (severity)* | |  |
| No COPD  Mild COPD  Moderate COPD | 201 (59.8%)  106 (31.6%)  29 (8.6%) | 143 (43.9%)  134 (41.1%)  49 (15.0%) |
|  |  |  |
| **Among non-smoking women** | Study controls  (n=326) | Study cases  (n=318) |
| Based on physician diagnosis and ATS criteria | |  |
| No pulmonary disease  Probably chronic bronchitis  Chronic bronchitis | 306 (93.9%)  20 (6.1%)  0 | 0  266 (83.6%)  52 (16.4%) |
| Based on spirometry by GOLD criteria (severity)* | |  |
| No COPD  Mild COPD  Moderate COPD | 198 (60.9%)  99 (30.5%)  28 (8.6%) | 138 (45.0%)  124 (40.4%)  45 (14.7%) |

* Missing data, n=12; there was 8 subjects with no pulmonary function data (1 in study controls and 7 in study cases) and 4 subjects with pulmonary function data but we can not categorize to severity (FEV1/FVC≥0.7, FEV1<0.3).

**eTable 3. Relationships between major variable of cooking oil fume exposure and other covariates in non-cook women and the risk of chronic bronchitis among non-smoking women (N=644).**

| **Variables** | **No pulmonary disease (N=306)** |  | **Probably chronic bronchitis (N=285)** | | |  |  | **Definite chronic bronchitis (N=53)** | | |  |
| --- | --- | --- | --- | --- | --- | --- | --- | --- | --- | --- | --- |
|  | **N (%)** |  | **N (%)** | **COR (95%CI)** | **AOR (95%CI)**^*^ |  |  | **N (%)** | **COR (95%CI)** | **AOR (95%CI)**^*^ |  |
| ***Excluding no cook*** | 284 |  | 272 |  |  |  |  | 48 |  |  |  |
| **Meals per week**  **1-13**  **14-20**  **21-31** | **85 (29.9)**  **94 (33.1)**  **105 (37.0)** |  | **55 (20.2)**  **84 (30.9)**  **133 (48.9)** | **1**  **1.38 (0.88-2.16)**  **1.96 (1.28-2.99)** | **1**  **1.52 (0.95-2.42)**  **2.14 (1.36-3.36)**^†^ |  |  | **5 (10.4)**  **14 (29.2)**  **29 (60.4)** | **1**  **2.53 (0.88-7.32)**  **4.69 (1.74-12.65)** | **1**  **2.71 (0.84-8.14)**^‡^  **4.73 (1.65-13.53)**^†^ |  |
| Age |  |  |  | 1.00 (0.98-1.02) | 0.99 (0.97-1.01) |  |  |  | 0.99 (0.96-1.02) | 0.96 (0.92-1.00) |  |
| Height |  |  |  | 1.02 (0.99-1.05) | 1.02 (0.99-1.06) |  |  |  | 1.00 (0.94-1.05) | 0.99 (0.94-1.05) |  |
| Education level^§^  Junior high school  Primary school  illiteracy | 34 (12.0)  165 (58.3)  84 (29.7) |  | 49 (18.0)  131 (48.2)  92 (33.8) | 1  0.57 (0.35-0.93)  0.78 (0.46-1.32) | 1  0.45 (0.26-0.77)  0.64 (0.34-1.19) |  |  | 7 (14.6)  24 (50.0)  17 (35.4) | 1  0.73 (0.29-1.82)  1.01 (0.39-2.65) | 1  0.38 (0.14-1.05)  0.60 (0.19-1.92) |  |
| Smoking status  Non-smoker  Second-hand smoker | 96 (33.8)  188 (66.2) |  | 72 (26.5)  200 (73.5) | 1  1.42 (0.99-2.04) | 1  1.41 (0.97-2.07) |  |  | 3 (6.3)  45 (93.8) | 1  7.66 (2.32-25.28) | 1  7.43 (2.22-24.87) |  |
| Burning incense  No  Yes | 106 (37.3)  178 (62.7) |  | 105 (38.6)  167 (61.4) | 1  0.95 (0.67-1.33) | 1  0.97 (0.67-1.39) |  |  | 13 (27.1)  35 (72.9) | 1  1.60 (0.82-3.17) | 1  1.57 (0.77-3.23) |  |
| Tea consumption  No  Yes | 218 (76.8)  66 (23.2) |  | 232 (85.3)  40 (14.7) | 1  0.57 (0.37-0.88) | 1  0.47 (0.29-0.75) |  |  | 46 (95.8)  2 (4.2) | 1  0.14 (0.03-0.61) | 1  0.10 (0.02-0.43) |  |

Abbreviation: AOR = adjusted OR; COR = crude OR; OR = odds ratio.

^*^Adjusting for age, second hand smoke status, height, education level, burning incense, and tea consumption.

^†^Trend test from 1-13, 14-20, to 21-31 meals per week in probably chronic bronchitis: p=0.009; and in chronic bronchitis: p=0.0023.

^‡^Trend test from no pulmonary disease, probably chronic bronchitis, to chronic bronchitis in 14-20 meals per week: p=0.020; and in 21-31 meals per week: p<0.0001.

^§^Missing data, n=1 in no pulmonary disease group.

**eTable 4**. **Relationships between cooking and ventilation conditions in home kitchens in 20-40 years old status and the severity of COPD according to GOLD criteria (N=632).**

| **Variables** | **No COPD (N=336)** | |  | **Mild+Moderate COPD (N=296)** | | |  |
| --- | --- | --- | --- | --- | --- | --- | --- |
|  | **N (%)** | |  | **N (%)** | **COR (95%CI)** | **AOR (95%CI)^*^** |  |
| *Cigarette smoking/SHS status* | | |  |  |  |  |  |
| Non-smoker  Second-hand smoker | | 108 (32.1)  228 (67.9) |  | 77 (26.0)  219 (74.0) | 1  1.35 (0.95-1.91) | 1  1.30 (0.91-1.86) |  |
|  | |  |  |  |  |  |  |
| Cooked in the kitchen  No  Daily | | 23 (6.9)  312 (93.1) |  | 12 (4.1)  282 (95.9) | 1  1.73 (0.85-3.55) | 1  1.34 (0.63-2.82) |  |
| ***Excluding no cook*** | |  |  |  |  |  |  |
| Age stared cooking  > 22 years  ≤ 22 years | | 139 (44.6)  173 (55.5) |  | 137 (48.6)  145 (51.4) | 1  0.85 (0.62-1.18) | 1  0.72 (0.51-1.02) |  |
| Meals per week  1-13  14-20  21-31 | | 85 (27.2)  100 (32.1)  127 (40.7) |  | 58 (20.6)  91 (32.3)  133 (47.2) | 1  1.33 (0.86-2.07)  1.54 (1.02-2.32) | 1  1.26 (0.80-1.97)  1.35 (0.87-2.08) |  |
| Fuel used for cooking^†^  Gas/electric stove  Biomass fuels  Coal | | 194 (62.4)  74 (23.8)  43 (13.8) |  | 158 (56.0)  73 (25.9)  51 (18.1) | 1  1.21 (0.82-1.78)  1.46 (0.92-2.30) | 1  0.89 (0.56-1.43)  1.20 (0.71-2.00) |  |
| Windows in the home kitchen^‡^  No  ≥ 1 | | 22 (7.2)  285 (92.8) |  | 16 (5.8)  258 (94.2) | 1  1.25 (0.64-2.42) | 1  1.39 (0.70-2.77) |  |
| Ventilation in the home kitchen^§^  Poor  Good | | 14 (4.5)  297 (95.5) |  | 15 (5.4)  265 (94.6) | 1  0.83 (0.39-1.76) | 1  0.88 (0.41-1.89) |  |
| Installed fume extractor^††^  No  Yes | | 131 (42.7)  176 (57.3) |  | 138 (50.0)  138 (50.0) | 1  0.74 (0.54-1.03) | 1  0.88 (0.60-1.30) |  |
| **Eye irritation during cooking**^‡‡^  **No**  **Yes** | | **277 (97.5)**  **7 (2.5)** |  | **234 (93.6)**  **16 (6.4)** | **1**  **2.71 (1.09-6.69)** | **1**  **2.68 (1.06-6.75)** |  |
| Smokiness during cooking^§§^  No  Yes | | 267 (93.7)  18 (6.3) |  | 223 (89.2)  27 (10.8) | 1  1.80 (0.96-3.35) | 1  1.67 (0.88-3.15) |  |

Abbreviation: AOR = adjusted OR; COPD = chronic obstructive pulmonary diseases; COR = crude OR; GOLD = Global Initiative for Chronic Obstructive Lung Disease; OR = odds ratio.

^*^Adjusting for age, second hand smoke status, height, education level, burning incense and tea consumption.

^†^Missing data=1 (1,0). ^‡^Missing data=13 (5,8). ^§^Missing data=3 (1,2). ^††^Missing data=11 (5,6). ^‡‡^Missing data=60 (28,32). ^§§^Missing data=59 (27,32).
